# Evaluation of interleukin-6 and its soluble receptor components sIL-6R and sgp130 as markers of inflammation in inflammatory bowel diseases

**DOI:** 10.1007/s00384-018-3069-8

**Published:** 2018-05-11

**Authors:** Susanna Nikolaus, Georg H. Waetzig, Sven Butzin, Monika Ziolkiewicz, Natalie Al-Massad, Florian Thieme, Ulf Lövgren, Birgitte B. Rasmussen, Torsten M. Reinheimer, Dirk Seegert, Philip Rosenstiel, Silke Szymczak, Stefan Schreiber

**Affiliations:** 1 Department of Internal Medicine I, University Hospital Schleswig-Holstein, Campus Kiel, Arnold-Heller-Strasse 3, 24105 Kiel, Germany; 2 CONARIS Research Institute AG, Kiel, Germany; 3 Institute of Clinical Chemistry, University Hospital Schleswig-Holstein, Campus Kiel, Kiel, Germany; 4 Ferring Pharmaceuticals A/S, Copenhagen, Denmark; 5 Institute of Clinical Molecular Biology, Kiel University, Kiel, Germany; 6 Institute of Medical Informatics and Statistics, Kiel University, Kiel, Germany

**Keywords:** Biomarkers, Interleukin-6, Inflammatory bowel diseases, Crohn’s disease, Ulcerative colitis

## Abstract

**Purpose:**

Interleukin-6 (IL-6) production and signalling are increased in the inflamed mucosa in inflammatory bowel diseases (IBD). As published serum levels of IL-6 and its soluble receptors sIL-6R and sgp130 in IBD are from small cohorts and partly contradictory, we systematically evaluated IL-6, sIL-6R and sgp130 levels as markers of disease activity in Crohn’s disease (CD) and ulcerative colitis (UC).

**Methods:**

Consecutive adult outpatients with confirmed CD or UC were included, and their disease activity and medication were monitored. Serum from 212 CD patients (815 measurements) and 166 UC patients (514 measurements) was analysed, and 100 age-matched healthy blood donors were used as controls.

**Results:**

IL-6 serum levels were significantly elevated in active versus inactive CD and UC, also compared with healthy controls. However, only a fraction of IBD patients showed increased serum IL-6. IL-6 levels ranged up to 32.7 ng/mL in active CD (> 5000-fold higher than in controls), but also up to 6.9 ng/mL in inactive CD. Increases in active UC (up to 195 pg/mL) and inactive UC (up to 27 pg/mL) were less pronounced. Associations between IL-6 serum levels and C-reactive protein concentrations as well as leukocyte and thrombocyte counts were observed. Median sIL-6R and sgp130 levels were only increased by up to 15%, which was considered of no diagnostic significance.

**Conclusions:**

Only a minority of IBD patients shows elevated IL-6 serum levels. However, in these patients, IL-6 is strongly associated with disease activity. Its soluble receptors sIL-6R and sgp130 do not appear useful as biomarkers in IBD.

**Electronic supplementary material:**

The online version of this article (10.1007/s00384-018-3069-8) contains supplementary material, which is available to authorized users.

## Introduction

The pleiotropic cytokine interleukin-6 (IL-6) plays an important role in inflammatory diseases and inflammatory carcinogenesis. Strategies for its inhibition have gained increasing interest due to the success of the anti-interleukin-6 receptor (IL-6R) antibody tocilizumab in rheumatologic diseases [1–4]. A significantly increased IL-6 production was reported in stimulated monocytes from patients with active inflammatory bowel disease (IBD) in comparison with samples from inactive disease phases or healthy control individuals [5, 6]. A general increase of IL-6 in patients with IBD and animal models has been reported in several studies [7–13].

IL-6 signals via two mechanisms, classic signalling and trans-signalling [1–4, 14]. In classic signalling, IL-6 binds to the specific cell membrane IL-6R expressed only on certain cell types, mainly leukocytes and hepatocytes, and the resulting IL-6/IL-6R complex recruits preformed dimers of the ubiquitously expressed IL-6 signal transducer glycoprotein 130 (gp130). In trans-signalling, IL-6 binds to soluble IL-6R (sIL-6R) formed by alternative splicing or, mainly, by proteolytic shedding of IL-6R from cell membranes. The resulting IL-6/sIL-6R trans-signalling complex acts like a different, more pro-inflammatory cytokine with a much broader target range, as it can theoretically activate gp130 signal transduction on every body cell, resulting in signalling patterns that are similar to but consequentially distinct from those of classic IL-6 signalling. In order to prevent such global trans-signalling, soluble forms of gp130 (sgp130) buffer the IL-6/sIL-6R complex in the blood. In the absence of inflammatory triggers, IL-6 is expressed in the low picogram range, whereas sIL-6R and sgp130 are expressed in the low to mid nanogram range (Supplemental Table 1). This mechanism indirectly also partly buffers IL-6 signalling as a whole [15], and even small congenital elevations of sIL-6R levels can reduce the risk for cardiovascular and autoimmune diseases [1, 4, 14].

There is good evidence that IL-6 levels, particularly in the context of IL-6 trans-signalling leading to activation of the pro-inflammatory signal transducer and activator of transcription-3 (STAT3), are strongly elevated in the inflamed mucosa in IBD [16–23]. IL-6 is the main inducer of C-reactive protein (CRP) [24], and IL-6 and sIL-6R levels have been shown by some studies to be positively associated with CRP levels in IBD [9, 11, 25]. A pilot phase II study using the neutralising anti-IL-6R antibody tocilizumab suggested a clinical response in Crohn’s disease (CD) patients [26], and several inhibitors of the IL-6 pathway are in clinical development, some also for IBD [3, 14, 27]. Multiple studies have reported significant increases in circulating IL-6 and sIL-6R in patients with IBD, albeit in different concentration ranges (Supplementary Table 1), as well as positive associations between IL-6 and sIL-6R serum levels and both activity indices and biomarkers of IBD [9, 11, 23, 25, 28, 29]. However, published levels and associations of biomarkers in IBD in general [30] and of IL-6, sIL-6R and sgp130 in particular are so diverse and partly contradictory (Supplementary Table 1) that a systematic analysis on a larger scale appeared warranted.

Therefore, the aim of the present study was to systematically analyse IL-6 and sIL-6R serum levels in a large all-comer cohort of outpatients with CD and ulcerative colitis (UC) and healthy control individuals. In addition, sgp130 levels were measured in a subset of patients and in all controls during the exploratory phase of the study. Multiple samplings and longitudinal follow-up in all available patients as well as a study duration of more than 1 year were used in this real-life, single-centre cohort to minimise bias and optimise association analyses with numerous physiological and pathophysiological parameters. Taken together, our study provides comprehensive data on the suitability and limitations of IL-6, sIL-6R and sgp130 as potential biomarkers in IBD.

## Materials and methods

### Patients and clinical data collection

In the IBD outpatient clinic of the Department of Internal Medicine I at the University Hospital Schleswig-Holstein (UKSH, Campus Kiel, Germany), consecutive adult patients with confirmed IBD were characterised and their disease activity as well as their medical treatment was monitored by regular visits, laboratory assessments of serum aliquots, clinical examinations, disease activity scores recorded at the time of blood collection (CD: Crohn’s Disease Activity Index (CDAI) [31] and Harvey-Bradshaw Index (HBI) [32]; UC: Colitis Activity Index (CAI) [33] and (Partial) Mayo Score [34]) as well as endoscopies. Diverse parameters were retrieved from the patients’ electronic hospital records and double-checked by at least two physicians. Serum from 100 age-matched healthy blood donors was obtained from the UKSH Department of Transfusion Medicine to serve as a control group. Ethics approval was granted by the ethics committee of the medical faculty of Kiel University prior to the study (AZ D489/14). The ethical concept of healthcare-embedded biobanking with broad informed consents as implemented in Kiel has recently been described [35]. Characteristics of IBD patients and healthy controls are summarised in Table 1.

**Table 1:** Characteristics of IBD patients and healthy controls

	Crohn’s disease	Ulcerative colitis	Controls
Number of individuals	212	166	100
Number of visits (longitudinal follow-up)	2 (1–24)^a^	2 (1–20)	1
Males	34.7%	45.1%	68.00%
Age at sampling [years]	37 (18–71)	42.5 (17–78)	44 (19–68)
Body mass index [kg/m^2^]	23.7 (14.7–53.6)	25.3 (16.8–48.3)	n.d.
Weight [kg]	70 (35–168.8)	78 (41–148)	n.d.
Smokers	48.2%	37.9%	n.d.
Time after diagnosis [years]	9 (0–40)	4.5 (0–41)	n.a.
Crohn’s Disease Activity Index (CDAI)	127.5 (0–583)	n.a.	n.a.
Harvey-Bradshaw Index (HBI)	3 (0–30)	n.a.	n.a.
Colitis Activity Index (CAI)	n.a.	5 (0–17)	n.a.
Mayo Score	n.a.	3 (0–11)	n.a.
Partial Mayo Score (PMS)	n.a.	2 (0–9)	n.a.
Aminosalicylate therapy	22.5%	91.0%	n.a.
Immunosuppressive therapy	14.1%	19.0%	n.a.
Monoclonal antibody therapy, except for anti-tumour necrosis factor-alpha (TNF-α) antibodies	15.9%	3.2%	n.a.
Steroid therapy	41.2%	63.6%	n.a.
Tumour necrosis factor-alpha (TNF-α) antagonist therapy	86.4%	54.1%	n.a.

*n.a.*, not applicable; *n.d.*, not determined

^a^All values in this format represent median (range)

### Measurement of IL-6, sIL-6R and sgp130

IL-6 and sIL-6R levels were determined in all patients and control samples. In the exploratory phase of the study, sgp130 levels were also determined in all control samples and in 212 patients with CD and 166 patients with UC, the ratio approximately reflecting the proportions of outpatients in our clinic. Quantikine® enzyme-linked immunosorbent assay (ELISA) kits (R&D Systems/Bio-Tecne; Wiesbaden, Germany) were used with a highly standardised protocol (for details, see Supplementary methods).

### Data analysis and statistics

All parameters investigated in the present study are listed in Supplementary Table 2. For details of the statistical methods, see Supplementary methods. Since more than 50% of the IL-6 measurements were below the detection limit of 3.13 pg/mL, we divided all IL-6 values of the complete dataset into three categories defined as (1) below detection limit, (2) between detection limit and third quartile and (3) larger than third quartile. We compared the two extreme groups 1 and 3 in all our analyses in order to clearly differentiate between patients with high IL-6 concentrations and patients with signals close to the detection limit. Concentrations of IL-6, sIL-6R and sgp130 were compared within patients, between patient groups and between patients and healthy controls. For each patient, we defined active and/or inactive time points based on either disease activity scores (active: CDAI > 150 points or CAI > 4 points; elevated (moderate to severe) activity: CDAI > 250 points or CAI > 6 points; inactive: CDAI ≤ 150 points or CAI ≤ 4 points) or CRP levels (active: CRP > 5 mg/L; inactive: CRP ≤ 5 mg/L). We used two different cohorts in our comparisons. First, we used all active and inactive time points from all patients (full dataset/cohort), and second, we selected only the most active (highest CDAI, CAI or CRP) and the most inactive time points (lowest CDAI, CAI or CRP) from those patients for whom both active and inactive time points were available (reduced dataset/cohort). Supplementary Table 3 shows the number of patients and measurements for the cohorts and subcohorts. Comparisons between active, inactive and healthy control samples were performed using a nonparametric bootstrap approach. Associations with clinical and serological parameters were analysed using all available data. For data presented in boxplots, the box corresponds to the interquartile range, the median is displayed as a bold line, the whiskers represent the most extreme values within the 1.5-fold of the interquartile range and values outside this range are displayed as single dots.

## Results

### IL-6, sIL-6R and sgp130 levels in the complete patient cohorts

**Fig. 1:**
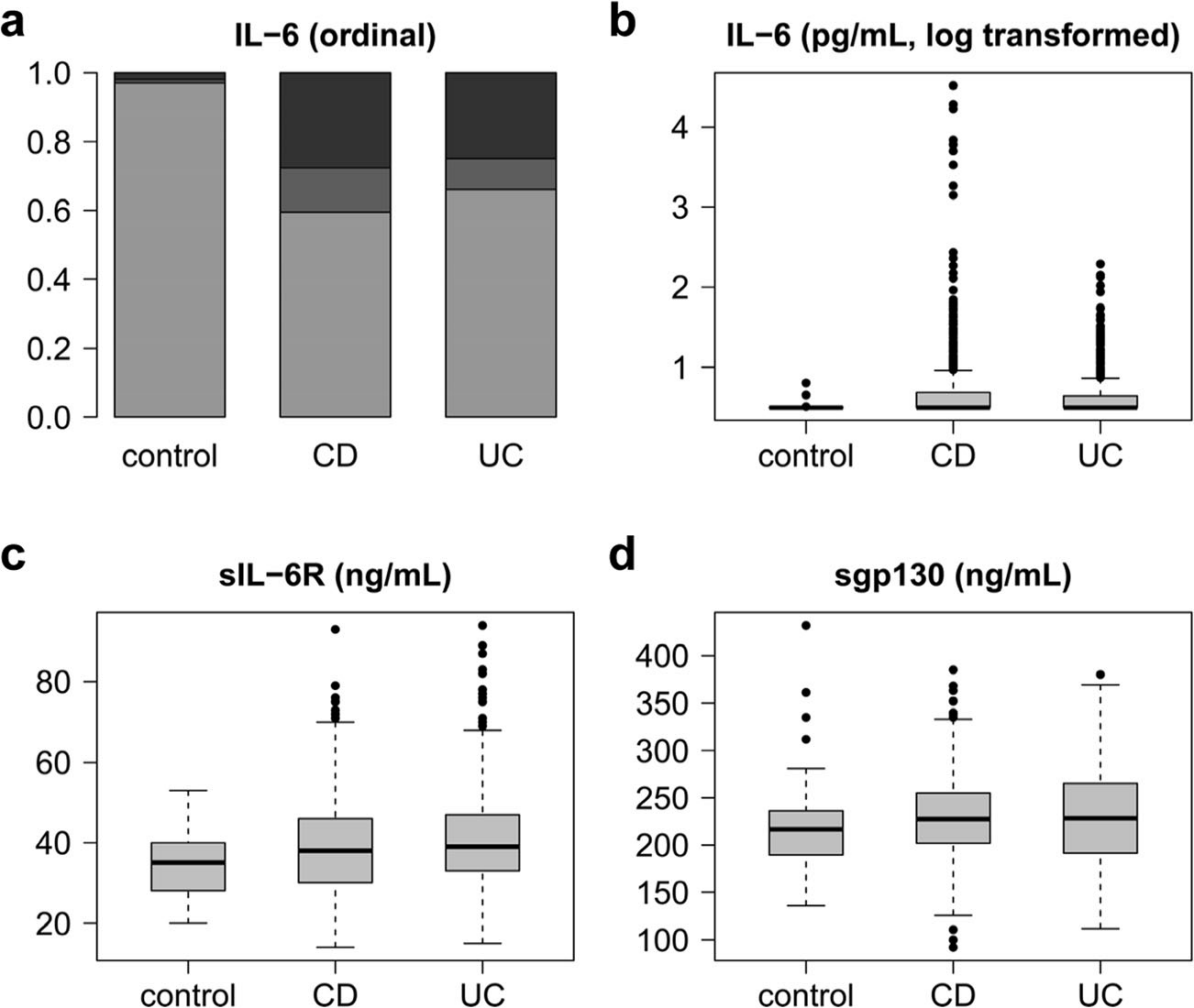
Overview of IL-6, sIL-6R and sgp130 serum levels in healthy controls and patients with Crohn’s disease (CD) or ulcerative colitis (UC). **a** Ordinal representation of IL-6 levels across all groups: below the detection limit of 3.13 pg/mL (light grey), between the detection limit and the third quartile (grey) and above the third quartile (black). **b** Logarithmic representation of IL-6 levels. **c** sIL-6R levels. **d** sgp130 levels

Table 2 summarises the measured serum levels of IL-6, sIL-6R and sgp130. Figure 1 shows the overall distribution of values. For the respective statistical data, see Supplementary Tables 4 and 5. As > 50% of IL-6 measurements in all groups were below the detection limit (≤ 3.13 pg/mL), ordinal IL-6 data were statistically evaluated by comparing the two most extreme groups (below detection limit vs top quartile). Figure 1a shows that the proportion of values above the detection limit is increased in IBD compared to healthy controls. Quantitative IL-6 data are presented on a logarithmic scale due to their extreme variation (Fig. 1b). While an increase in variation compared to normal controls was also observed in CD and UC patients for both sIL-6R (Fig. 1c) and sgp130 (Fig. 1d), the increase of their median levels was less than 15% and not consistent with regard to disease activity (Fig. 1c, d; Table 2).

**Table 2:** Circulating levels of IL-6, sIL-6R and sgp130

Groups	IL-6 (pg/mL)	sIL-6R (ng/mL)	sgp130 (ng/mL)
Controls	≤ 3^a^ (≤ 3–6) (*n* = 100)	35 (20–53) (*n* = 100)	217 (136–432) (*n* = 100)
Inactive CD	≤ 3 (≤ 3–6872)^b^ (*n* = 458)^d^	40 (14–93)^b^ (*n* = 456)	227 (92–368) (*n* = 228)
Active CD	≤ 3 (≤ 3–32,671)^b, c^ (*n* = 328)	36 (16–72)^c^ (*n* = 325)	232 (140–385)^b^ (*n* = 135)
Inactive UC	≤ 3 (≤ 3–27)^b^ (*n* = 222)	39 (19–87)^b^ (*n* = 222)	197 (112–306) (*n* = 32)
Active UC	≤ 3 (≤ 3–195)^b, c^ (*n* = 273)	39 (15–83)^b^ (*n* = 273)	249 (115–341) (*n* = 41)

All values represent median (range) and were rounded to the nearest integer. For a comparison with reports in the literature, please see Supplementary Table 1

^a^Detection limit of the IL-6 ELISA, 3.13 pg/mL

^b^*p* < 0.05 or less versus healthy controls

^c^*p* < 0.05 or less for active (CDAI > 150 or CAI > 4) versus inactive disease (CDAI ≤ 150 or CAI ≤ 4)

^d^Number of samples from different visits. IL-6 and sIL-6R were measured in a total of 212 patients with CD and 166 patients with UC; sgp130 was measured in 81 patients with CD and 22 patients with UC

**Fig. 2:**
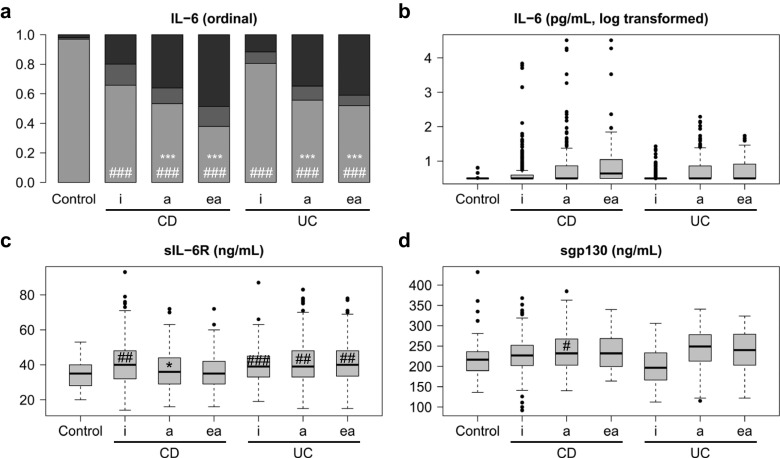
IL-6, sIL-6R and sgp130 serum levels in healthy controls and in all patients with Crohn’s disease (CD) or ulcerative colitis (UC) differentiated according to disease activity scores. **a** Ordinal representation of IL-6 levels across all groups: below the detection limit of 3.13 pg/mL (light grey), between the detection limit and the third quartile (grey) and above the third quartile (black). For significance analyses, the two extreme groups (below the detection limit and above the third quartile) were compared. **b** Logarithmic representation of IL-6 levels. **c** sIL-6R levels. **d** sgp130 levels. a, active disease (Crohn’s Disease Activity Index (CDAI) > 150 or Colitis Activity Index (CAI) > 4); ea, elevated (moderate to severe) disease activity (CDAI > 250 or CAI > 6); i, inactive disease (CDAI ≤ 150 or CAI ≤ 4). Significant differences between active and inactive disease: *, *p* < 0.05; **, *p* < 0.01; ***, *p* < 0.001. Significant differences between active or inactive disease and controls: #, *p* < 0.05; ##, *p* < 0.01; ###, *p* < 0.001

**Fig. 3:**
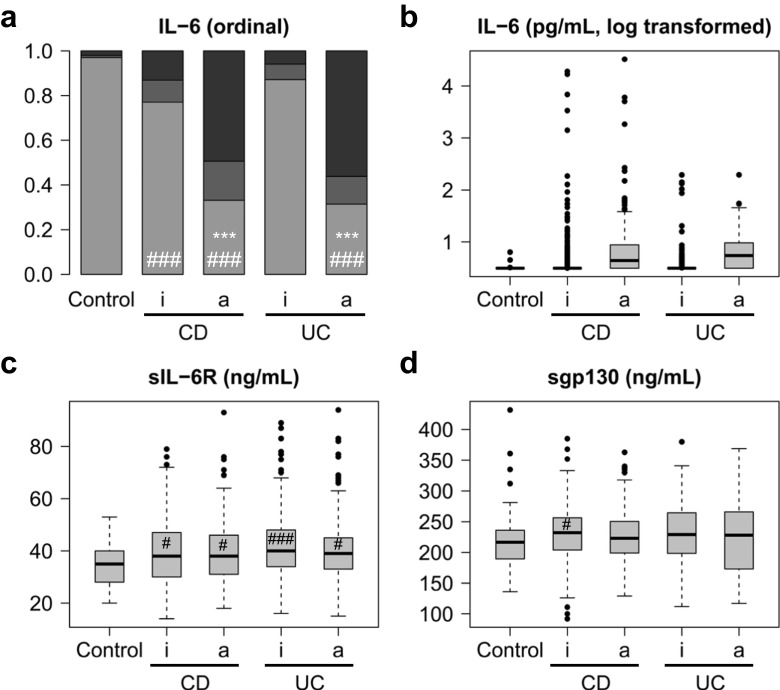
IL-6, sIL-6R and sgp130 serum levels in healthy controls and in all patients with Crohn’s disease (CD) or ulcerative colitis (UC) differentiated according to C-reactive protein (CRP) levels. **a** Ordinal representation of IL-6 levels across all groups: below the detection limit of 3.13 pg/mL (light grey), between the detection limit and the third quartile (grey) and above the third quartile (black). For significance analyses, the two extreme groups (below the detection limit and above the third quartile) were compared. **b** Logarithmic representation of IL-6 levels. **c** sIL-6R levels. **d** sgp130 levels. a, active disease (CRP > 5 mg/L); i, inactive disease (CRP ≤ 5 mg/L). Significant differences between active and inactive disease: *, *p* < 0.05; **, *p* < 0.01; ***, *p* < 0.001. Significant differences between active or inactive disease and controls: #, *p* < 0.05; ##, *p* < 0.01; ###, *p* < 0.001

When patient subgroups were analysed based on their disease activity scores (Fig. 2) or CRP levels (Fig. 3), elevated IL-6 levels were significantly associated with disease activity in both CD and UC. For the respective statistical data, see Supplementary Tables 6 and 7. This association was more pronounced with CRP than with disease activity scores, particularly in UC (cf. Figs. 2a and 3a). A subgroup of patients with elevated (moderate to severe) disease activity scores (“ea” in Fig. 2; CDAI > 250 or CAI > 6) showed higher IL-6 levels than all active patients, including those with mild activity, taken together (“a” in Fig. 2; CDAI > 150 or CAI > 4). Nevertheless, the majority also of patients with active CD or UC did not show elevated IL-6 levels (Table 2; Fig. 2a). The minor increase in sIL-6R levels was independent of disease activity in both CD and UC (Figs. 2c and 3c) and, as with IL-6, was more pronounced when patients were grouped according to CRP levels (Fig. 3c).

### IL-6, sIL-6R and sgp130 levels in subcohorts with matched measurements

**Fig. 4:**
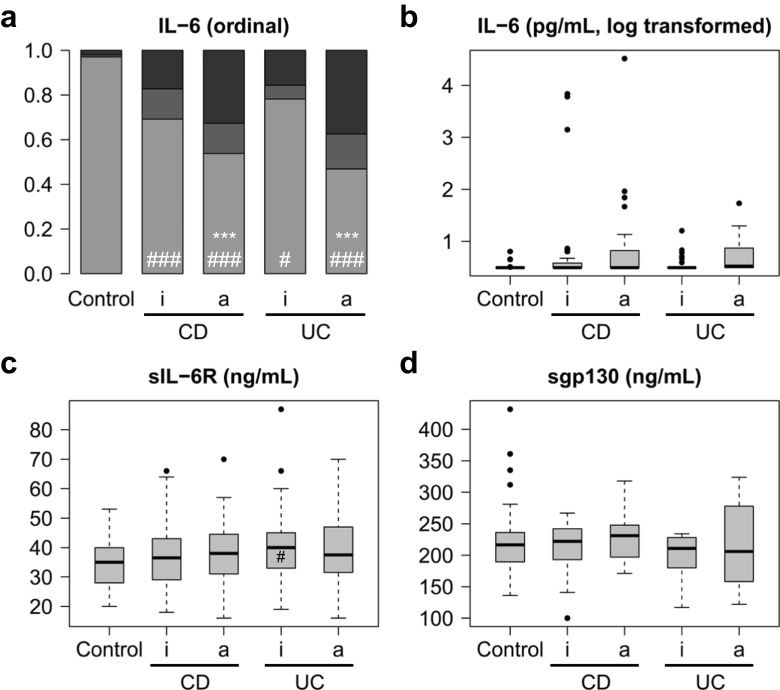
IL-6, sIL-6R and sgp130 serum levels in healthy controls and in selected patients with Crohn’s disease (CD) or ulcerative colitis (UC) differentiated according to disease activity scores. Only patients with at least one time point with active and one with inactive disease were included. If more than one time point per condition was available, only data from the two most extreme time points (highest and lowest disease activity score) were included. **a** Ordinal representation of IL-6 levels across all groups: below the detection limit of 3.13 pg/mL (light grey), between the detection limit and the third quartile (grey) and above the third quartile (black). For significance analyses, the two extreme groups (below the detection limit and above the third quartile) were compared. **b** Logarithmic representation of IL-6 levels. **c** sIL-6R levels. **d** sgp130 levels. a, active disease (Crohn’s Disease Activity Index (CDAI) > 150 or Colitis Activity Index (CAI) > 4); i, inactive disease (CDAI ≤ 150 or CAI ≤ 4). Significant differences between active and inactive disease: *, *p* < 0.05; **, *p* < 0.01; ***, *p* < 0.001. Significant differences between active or inactive disease and controls: #, *p* < 0.05; ##, *p* < 0.01; ###, *p* < 0.001

**Fig. 5:**
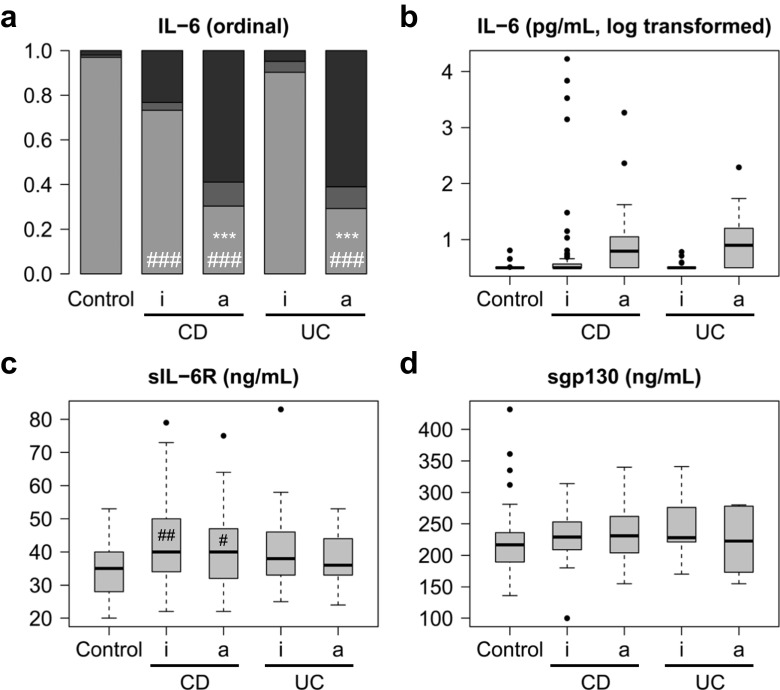
IL-6, sIL-6R and sgp130 serum levels in healthy controls and in selected patients with Crohn’s disease (CD) or ulcerative colitis (UC) differentiated according to C-reactive protein (CRP) levels. Only patients with at least one time point with CRP > 5 mg/L and one time point with CRP ≤ 5 mg/L were included. If more than one time point per condition was available, only data from the two most extreme time points (highest and lowest CRP levels) were included. **a** Ordinal representation of IL-6 levels across all groups: below the detection limit of 3.13 pg/mL (light grey), between the detection limit and the third quartile (grey) and above the third quartile (black). For significance analyses, the two extreme groups (below the detection limit and above the third quartile) were compared. **b** Logarithmic representation of IL-6 levels. **c** sIL-6R levels. **d** sgp130 levels. a, active disease (CRP > 5 mg/L); i, inactive disease (CRP ≤ 5 mg/L). Significant differences between active and inactive disease: *, *p* < 0.05; **, *p* < 0.01; ***, *p* < 0.001. Significant differences between active or inactive disease and controls: #, *p* < 0.05; ##, *p* < 0.01; ###, *p* < 0.001

In order to investigate whether the large number of patients with inactive disease and the multiple visits per patient (in which disease activity was eventually controlled by anti-inflammatory drugs) confounded conclusions drawn from the complete dataset, a reduced dataset for patients with CD and UC was analysed (Supplementary Table 3). For this purpose, only patients with at least one time point with active and one time point with inactive disease were included. If more than one time point per condition was available, the most contrasting data (highest and lowest disease activity scores (Fig. 4) or CRP levels (Fig. 5)) were analysed. For the respective statistical data, see Supplementary Tables 8 and 9. The results of this subcohort analysis did not differ much from the results obtained with the full dataset (Figs. 4 and 5; cf. Figs. 2 and 3). While significantly higher IL-6 serum levels were observed in active versus inactive disease, there were still many patients with active disease and normal IL-6 levels, regardless of whether activity was defined by disease score or CRP. Also in this subcohort, sIL-6R and gp130 levels were only slightly increased.

Taken together, a consistent, both biologically and statistically significant, increase was observed only for IL-6, but not for sIL-6R or sgp130. However, while this increase in IL-6 was seen on the population level, it was less than 50% of individuals with active disease according to scores who contributed to the signal (Figs. 2a and 4a). In contrast, a majority of CD and UC patients with elevated CRP levels showed IL-6 levels above the detection limit (Figs. 3a and 5a).

### Association of IL-6, sIL-6R and sgp130 levels with general, clinical and laboratory parameters

**Fig. 6:**
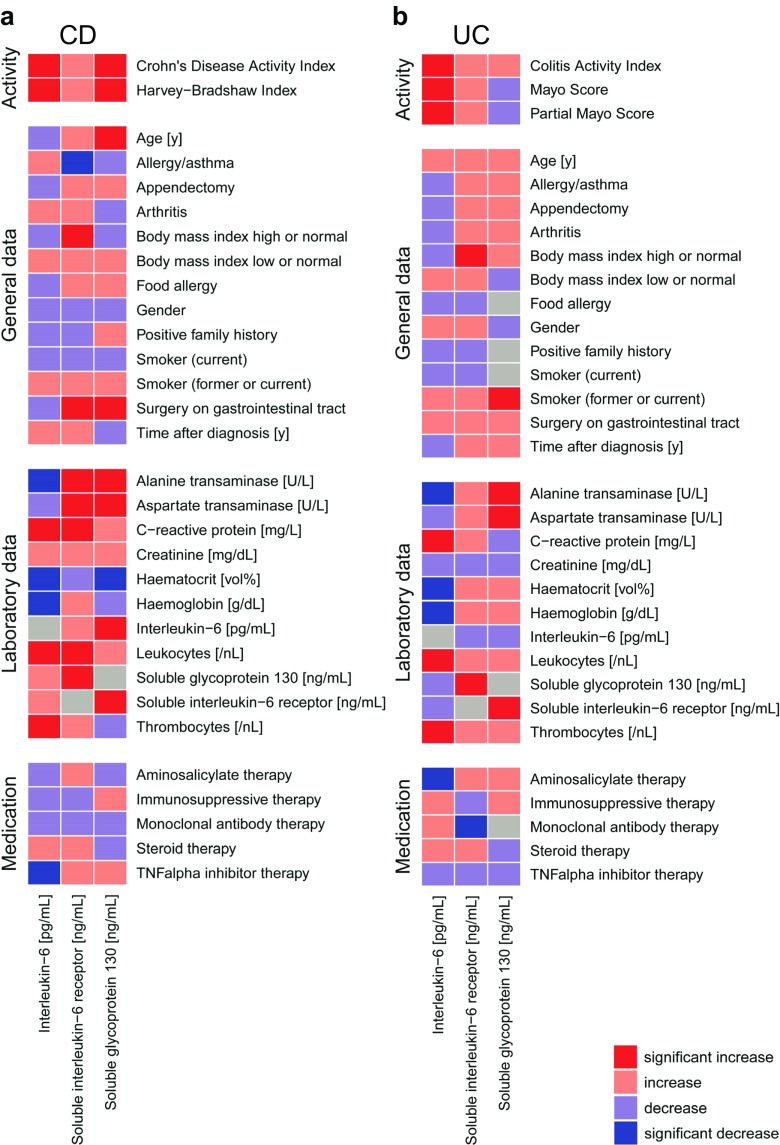
Association heat maps for Crohn’s disease (CD; **a**) and ulcerative colitis (UC; **b**). Tendentially or significantly positive (increase) or negative (decrease) associations of ordinal interleukin-6, soluble interleukin-6 receptor or soluble glycoprotein 130 serum levels with other parameters are visualised by colour code. For the complete data and *p* values, see Supplementary Tables 10, 11 and 12. Grey fields indicate parameters with only one characteristic value or self-associations

A wide spectrum of parameters was analysed for associations (Fig. 6; Supplementary Tables 2, 10, 11, 12 and 13). Disease activity-associated laboratory parameters were consistently positively (CRP, leukocytes and thrombocytes) or negatively (haematocrit and haemoglobin) associated with IL-6 levels in both CD and UC (Fig. 6a, b; Supplementary Table 10). There was no clear association between IL-6 and sIL-6R or sgp130 (Fig. 6a, b; Supplementary Tables 10, 11 and 12), but the slightly increased IL-6 buffer consisting of sIL-6R and sgp130 in CD and UC (Figs. 1, 2, 3, 4 and 5; Table 2) was mirrored by significant positive associations between these soluble receptor components (Fig. 6a, b; Supplementary Tables 11 and 12). In CD, but not in UC, sIL-6R and sgp130 levels were also positively associated with previous surgery on the gastrointestinal tract and suggestively (sIL-6R) or significantly (sgp130) negatively associated with haematocrit (Fig. 6a, b; Supplementary Tables 11 and 12). Regarding disease location, IL-6 was only positively associated with terminal ileitis in CD, while sIL-6R and sgp130 showed a complex association with several locations, but rather small effect sizes (Supplementary Table 13). In UC, no significant associations with disease location were observed (Supplementary Table 13). The use of tumour necrosis factor-alpha (TNF-α) antagonists was significantly associated with reduced IL-6 levels in CD (Fig. 6a), whereas the same was observed for aminosalicylates in UC (Fig. 6b).

## Discussion

The main objective of the present study was to explore IL-6, sIL-6R and sgp130 as potential biomarkers in IBD on the background of conflicting prior reports (Supplemental Table 1) [7–13]. Previous publications demonstrating an overall increase in IL-6 levels in patients with IBD, particularly in those with active disease [7–11, 13], were confirmed by our findings. Broken down to the individual level, the signal was carried by less than 50% of patients with active disease: in contrast to previous smaller studies [7, 10, 11], median IL-6 levels were neither elevated in inactive nor in active disease (based on disease scores) compared to healthy control individuals, and the range of IL-6 levels was much wider [7, 10, 11]. Disease activity based on CRP was more strongly matched to elevated IL-6 levels than score-based activity, which is plausible due to the key role of IL-6 in CRP secretion.

Serum levels of IL-6 do not necessarily reflect the situation in the intestinal mucosa, where high local levels of IL-6 have been detected during inflammation [16–23], because blood circulation from the intestine through the liver may clear large amounts of IL-6. Similar to IL-6 in the present study, TNF-α serum levels are also not consistently found to be elevated in patients with IBD [36–40], despite the proven strong increase in mucosal TNF-α-producing cells and TNF-α levels in stool [16, 41–45] and the well-established efficacy of anti-TNF-α antibodies in CD and UC [46].

sIL-6R levels were only slightly increased in IBD patients. Our data confirm the findings of the small pilot study of Gustot et al. [11] and the general consensus on sIL-6R levels in health and chronic inflammation in the literature [1], whereas Nancey et al. [13] and particularly Mitsuyama et al. [9] measured higher overall levels. A tendency towards higher serum levels of sgp130 was observed in the present study for both active CD and UC, but no significant difference was observed between active and inactive patients. Again, our data confirm those by Gustot et al. [11] and the general consensus on sgp130 levels [1], whereas much less sgp130 was detected by Nancey et al. [13]. The reasons for the variance between these reports remain unclear, but the biological significance of both sIL-6R and sgp130 level changes appears to be limited in IBD.

We observed associations of IL-6, sIL-6R and sgp130 serum levels with general, clinical and laboratory parameters that largely confirm previous reports and are plausible with regard to the course of the diseases, including a positive association of IL-6 levels with disease activity, CRP levels as well as leukocyte and platelet counts. The use of TNF-α antagonists in CD and the use of aminosalicylates in UC were therapies for which we detected a significant reduction of IL-6 levels. The relevance of other associations is unclear: for example, the significant negative association between serum levels of the liver enzymes AST and ALT and the circulating components of the IL-6 system may reflect the abnormal liver function tests reported in IBD [47–53]. Some statistically significant associations also do not appear relevant due to their rather small effect sizes, such as the associations with disease location.

In summary, the present study shows that sIL-6R or sgp130 are not useful as biomarkers in IBD and that only a fraction of patients with active CD or UC have elevated IL-6 serum levels. It remains unexplored whether the individual differences reflect local IL-6 levels in the inflamed mucosa, too, or whether these are increased in the vast majority of patients. As a general biomarker of IBD disease activity, IL-6 adds little to established biomarkers like CRP, which, however, also have significant limitations. Taken together, IL-6 serum levels are another imperfect biomarker for IBD activity but may be useful for identifying patients with a particularly strong IL-6 disease signature in order to increase the likelihood of treatment success with drugs targeting IL-6 or its signalling pathways.

## Electronic supplementary material

ESM 1 (PDF 391 kb)
