# Supplementary material for: Evaluation of interleukin-6 and its soluble receptor components sIL-6R and sgp130 as markers of inflammation in inflammatory bowel diseases
Source: Int J Colorectal Dis. 2018 May 11;33(7):927–36. doi: 10.1007/s00384-018-3069-8 (PMC6002455; doi:10.1007/s00384-018-3069-8)
Supplement: Supplementary file 1 — (PDF 391 kb) [file 384_2018_3069_MOESM1_ESM.pdf]

SUPPLEMENTARY MATERIAL

Journal:

International Journal of Colorectal Disease

Article:

Evaluation of Interleukin-6 and its Soluble Receptor Components sIL-6R and sgp130 as Markers of Inflammation in Inflammatory Bowel Diseases

Authors:

Susanna Nikolaus,[1,*] Georg H. Waetzig,[2,*] Sven Butzin,[1] Monika Ziolkiewicz,[1] Natalie Al-Massad,[1] Florian Thieme,[3] Ulf Lövgren,[4] Birgitte B. Rasmussen,[4] Torsten M. Reinheimer,[4] Dirk Seegert,[2] Philip Rosenstiel,[5] Silke Szymczak,[6] Stefan Schreiber[1,5]

[1] Department of Internal Medicine I, University Hospital Schleswig-Holstein, Campus Kiel, Kiel, Germany;
[2] CONARIS Research Institute AG, Kiel, Germany;
[3] Institute of Clinical Chemistry, University Hospital Schleswig-Holstein, Campus Kiel, Kiel, Germany;
[4] Ferring Pharmaceuticals A/S, Copenhagen, Denmark;
[5] Institute of Clinical Molecular Biology, Kiel University, Kiel, Germany;
[6] Institute of Medical Informatics and Statistics, Kiel University, Kiel, Germany.

* These two authors contributed equally to this work.

Correspondence to:

Professor Stefan Schreiber
Department of Internal Medicine I
Kiel University and University Hospital Schleswig-Holstein, Campus Kiel
Arnold-Heller-Strasse 3
24105 Kiel, Germany
e-mail: s.schreiber@mucosa.de
phone: +49 431 500-22200
fax: +49 431 500-22204.

SUPPLEMENTARY METHODS

Measurement of IL-6, sIL-6R and sgp130

Upon the first thawing of serum aliquots prepared for this purpose, all measurements were performed immediately and in parallel using the following Quantikine® enzyme-linked immunosorbent assay (ELISA) kits (R&D Systems / Bio-Tecne; Wiesbaden, Germany): D6050 (human IL-6), DR600 (human sIL-6R) and, for all controls and a subgroup of IBD patients during the exploratory phase of the study, DGP00 (human sgp130). The analyses were performed exactly as specified in the manufacturer's manuals (IL-6: version 01.06, 749909.6, 11/13; sIL-6R: version 04.93, 750572.5, 10/14; sgp130: version 11.95, 750156.4, 4/14). Samples were analysed in duplicate and using the standards supplied by the manufacturer. Only measurements in the standard range were used for the analyses, outlier samples were re-tested using dilutions as specified by the manufacturer. According to the manufacturer, the ELISA D6050 detects IL-6 both unbound and bound to sIL-6R. Acquisition of colorimetric data was performed using an OPSYS MR microplate reader with Revelation Quicklink software (Dynex Technologies; Denkendorf, Germany).

Data analysis and statistics

Associations with clinical and serological parameters were analysed using all available data. To account for the multiple observations per patient, univariate mixed models with the independent variable (i.e. clinical parameter) as fixed effect and the patient identity code as random effect were used. Linear mixed models were fitted for the continuous variables sIL-6R and sgp130, whereas logistic regression models were estimated for the ordinal variable IL-6. For all mixed model analyses, all independent continuous variables were dichotomized based on the median.

Comparisons between active, inactive and healthy control samples were performed using a nonparametric bootstrap approach. Sampling with replacement was performed on the proband level, and a suitable test statistic was calculated on each bootstrap sample. Confidence intervals were estimated using the 2.5% and 97.5% quantiles of the distribution of the bootstrap test statistics. P-values were determined by 2* the proportion of bootstrap test statistics larger or smaller than zero, depending on the sign of the test statistic based on the original sample. As test statistics, we used the difference in medians or the median of the differences for the continuous variables sIL-6R and sgp130 in the unpaired or paired comparisons. For IL-6, the difference in proportions or the proportion of disagreement were applied.

All statistical tests were carried out two-sided using a nominal significance level of 5%. Analyses were performed with the statistical software R (version 3.3.1). R packages nlme (version 3.1-128) and MuMIn (version 1.15.6) were used to estimate linear mixed models and the variance explained by the fixed effect (= marginal R2). The presentation of data in boxplots was performed using the standard settings in R: the box corresponds to the interquartile range, the median is displayed as a bold line, the whiskers represent the most extreme values within the 1.5-fold of the interquartile range, and values outside this range are displayed as single dots.

SUPPLEMENTARY TABLES

Supplementary Table 1: Published circulating levels of IL-6, sIL-6R and sgp130 in patients with inflammatory bowel diseases

Publication	Groups	IL-6 (pg/mL)	sIL-6R (ng/mL)	sgp130 (ng/mL)	Selected associations ($p < 0.05$)[a]
Mahida *et al.* 1991 [1]	Healthy controls (n = 16)	<20 (<20–30) [median (range)]	n.d.	n.d.	CD: (–) IL-6 & albumin; no association with CRP.
	Active CD (n = 21)	47 (<20–250)	n.d.	n.d.	
	Active UC (n = 20)	<20 (<20–80)	n.d.	n.d.	
Hyams *et al.* 1993 [2]	Healthy controls (n = 29)	0.4 ± 0.2 [mean ± SEM]	n.d.	n.d.	Significant association between IL-6 levels and disease activity in both CD and UC.
	Pediatric CD (n = 49)	4 ± 1[b]	n.d.	n.d.	
	Pediatric UC (n = 26)	1 ± 0.3[b]	n.d.	n.d.	
Mitsuyama *et al.* 1995 [3]	Healthy controls (n = 69)	n.d. [mean ± SEM]	107 ± 2	n.d.	CD: (+) IL-6 & CRP; (+) IL-6 & platelet counts; (+) sIL-6R & CRP. UC: (+) IL-6 & CRP; (+) IL-6 & leukocyte counts; (+) sIL-6R & CRP.
	Inactive CD (n = 9)	5 ± 4	114 ± 7	n.d.	
	Active CD (n = 15)	16 ± 12[c]	142 ± 9[b,c]	n.d.	
	Inactive UC (n = 16)	4 ± 1	116 ± 7	n.d.	
	Active UC (n = 16)	10 ± 9[c]	148 ± 5[b,c]	n.d.	
Reinisch *et al.* 1999 [4]	Healthy controls (n = 32)	<1.3 (<1.3) [median (range)]	n.d.	n.d.	(+) IL-6 & disease activity (in primarily inflammatory CD patients); (+) IL-6 & CRP; (+) IL-6 & oromucosoid.
	Inactive CD (n = 44)	1.3 (1.3–8)[b]	n.d.	n.d.	
	Mod. active CD (n = 44)[d]	3 (1.3–35)[b,c]	n.d.	n.d.	
	Highly active CD (n = 48)	6 (1.3–35)[b,c]			
Gustot *et al.* 2005 [5]	Healthy controls (n = 15)	2 (0–4) [median (range)]	33 (21–50)	273 (213–375)	CD: (+) IL-6 & CRP; (+) sIL-6R & CRP; (–) sgp130 & CRP. UC: (–) sgp130 & CRP.
	Inactive CD (n = 20)	3 (0–7)	29 (20–50)	278 (229–449)	
	Active CD (n = 30)	15 (0–69)[b,c]	38 (31–56)[c]	220 (138–310)[b,c]	
	Inactive UC (n = 11)	6 (0–12)	35 (21–59)	265 (182–437)	
	Active UC (n = 13)	21 (0–293)[b,c]	37 (22–72)	176 (96–374)[b,c]	
Mitsuyama *et al.* 2006 [6]	Healthy controls (n = 17)	n.d. (only paired analyses with selected samples)	n.d. (see IL-6)	43 (27–59) [median (IQR)]	n.d.
	Inactive CD (n = 10)	n.d.	n.d.	63 (44–83)	
	Active CD (n = 13)	n.d.	n.d.	66 (44–88)	
	Inactive UC (n = 13)	n.d.	n.d.	81 (25–137)[b]	
	Active UC (n = 14)	n.d.	n.d.	94 (26–125)[b]	
Nancey *et al.* 2008 [7]	Healthy controls (n = 20)	3 ± 1 [mean ± SEM]	58 ± 6	n.d.	(+) IL-6 & CRP, but not sIL-6R & CRP.
	Inactive CD (n = 50)	50 ± 4[b]	82 ± 5[b]	n.d.	
	Active CD (n = 44)	80 ± 9[b,c]	77 ± 5[b]	n.d.	

Legend to Supplementary Table 1:

Data shown are from selected publications.
All numbers above 1 were rounded to the nearest integer.
[a]Positive (+) or negative (–) associations between the indicated parameter combinations.
[b]$p < 0.05$ or less vs healthy controls.
[c]$p < 0.05$ or less vs inactive disease.
[d]Moderately active: Crohn's Disease Activity Index (CDAI) 150–250; highly active: CDAI >250.
CD, Crohn's disease; IL-6, interleukin-6; IQR, interquartile range; n, number of individuals per group; n.d., not determined; SEM, standard error of the mean; sgp130, soluble gp130; sIL-6R, soluble interleukin-6 receptor; UC, ulcerative colitis.

References for Supplementary Table 1:

[1] Mahida YR, Kurlac L, Gallagher A, Hawkey CJ (1991) High circulating concentrations of interleukin-6 in active Crohn's disease but not ulcerative colitis. Gut 32:1531-1534.
[2] Hyams JS, Fitzgerald JE, Treem WR, Wyzga N, Kreutzer DL (1993) Relationship of functional and antigenic interleukin 6 to disease activity in inflammatory bowel disease. Gastroenterology 104:1285-1292.
[3] Mitsuyama K, Toyonaga A, Sasaki E, Ishida O, Ikeda H, Tsuruta O, Harada K, Tateishi H, Nishiyama T, Tanikawa K (1995) Soluble interleukin-6 receptors in inflammatory bowel disease: relation to circulating interleukin-6. Gut 36:45-49.
[4] Reinisch W, Gasche C, Tillinger W, Wyatt J, Lichtenberger C, Willheim M, Dejaco C, Waldhor T, Bakos S, Vogelsang H, Gangl A, Lochs H (1999) Clinical relevance of serum interleukin-6 in Crohn's disease: single point measurements, therapy monitoring, and prediction of clinical relapse. Am J Gastroenterol 94:2156-2164.
[5] Gustot T, Lemmers A, Louis E, Nicaise C, Quertinmont E, Belaiche J, Roland S, Van Gossum A, Deviere J, Franchimont D (2005) Profile of soluble cytokine receptors in Crohn's disease. Gut 54:488-495.
[6] Mitsuyama K, Tomiyasu N, Suzuki A, Takaki K, Takedatsu H, Masuda J, Yamasaki H, Matsumoto S, Tsuruta O, Toyonaga A, Sata M (2006) A form of circulating interleukin-6 receptor component soluble gp130 as a potential interleukin-6 inhibitor in inflammatory bowel disease. Clin Exp Immunol 143:125-131.
[7] Nancey S, Hamzaoui N, Moussata D, Graber I, Bienvenu J, Flourie B (2008) Serum interleukin-6, soluble interleukin-6 receptor and Crohn's disease activity. Dig Dis Sci 53:242-247.

Supplementary Table 2: General, clinical and laboratory parameters

Data group	Parameter
General data	Age at sampling [y]
	Allergy/asthma
	Appendectomy
	Arthritis as a comorbidity
	Body mass index (BMI), sorted into overlapping groups:
	group 1: high (BMI ≥ 25) or normal (18.5 ≤ BMI < 25)
	group 2: low (< 18.5) or normal (18.5 ≤ BMI < 25)
	Food allergy
	Gender
	Positive family history of inflammatory bowel diseases
	Smoker (current)
	Smoker (former or current)
	Surgery on gastrointestinal tract (ever)
	Time after diagnosis [y]
Activity (CD)	Crohn's Disease Activity Index (CDAI)
	Harvey-Bradshaw Index (HBI)
Activity (UC)	Colitis Activity Index (CAI)
	Mayo Score
	Partial Mayo Score (PMS)
Laboratory data	Alanine transaminase (ALT; formerly glutamic-pyruvic transaminase, GPT) [U/L]
	Aspartate transaminase (AST; formerly glutamic-oxaloacetic transaminase, GOT) [U/L]
	C-reactive protein (CRP) [mg/L]
	Creatinine [mg/dL]
	Haematocrit [vol%]
	Haemoglobin [g/dL]
	Interleukin-6 [pg/mL]
	Leukocytes [/nL]
	Soluble glycoprotein 130 [ng/ml]
	Soluble interleukin-6 receptor [ng/ml]
	Thrombocytes [/nL]
Medication	Aminosalicylate therapy
	Immunosuppressive therapy
	Monoclonal antibody therapy, except for anti-tumour necrosis factor-alpha (TNF-α) antibodies
	Steroid therapy
	TNF-α antagonist therapy

Supplementary Table 3: Numbers of patients and measurements per cohort and subcohort

Group	CD: no. of patients	CD: no. of measurements	UC: no. of patients	UC: no. of measurements
All	212	815	166	514
All: activity by score	209	786	163	495
All: activity by CRP	212	814	166	514
Subset: activity by score	52	104	32	64
Subset: activity by CRP	56	112	41	82

CD, Crohn's disease; CRP, C-reactive protein; no., number; UC, ulcerative colitis. Note that in a few cases, not both the activity score [CD: Crohn's Disease Activity Index (CDAI); UC: Colitis Activity Index (CAI)] and the CRP level had been obtained on each visit.

Supplementary Table 4: Statistical data for Figure 1a

**Ordinal groups for interleukin-6**	**Control**	**Crohn's disease**	**Ulcerative colitis**
Values < detection limit (3.13 pg/mL)	0.97	0.59	0.66
detection limit ≤ values ≤ 3rd quartile	0.01	0.13	0.09
3rd quartile < values	0.02	0.28	0.25

Supplementary Table 5: Statistical data for Figure 1b–d

**Variables**	**Control**	**Crohn's disease**	**Ulcerative colitis**
Interleukin-6 (logarithmic; pg/mL)	0.5 (0.5–0.8)	0.5 (0.5–4.51)	0.5 (0.5–2.29)
Soluble interleukin-6 receptor (ng/mL)	35 (20–53)	38 (14–93)	39 (15–87)
Soluble glycoprotein 130 (ng/mL)	217 (136–432)	228 (92–385)	229 (112–341)

All values represent median (range) and were rounded to the nearest integer.

Supplementary Table 6: Statistical data for Figure 2

		**Active vs controls**			**Inactive vs controls**			**Inactive vs active**			**Enhanced activity vs controls**			**Inactive vs enhanced activity**		
**Var.**	**Dis.**	**Est.**	**95% CI**	***p***	**Est.**	**95% CI**	***p***	**Est.**	**95% CI**	***p***	**Est.**	**95% CI**	***p***	**Est.**	**95% CI**	***p***
IL-6 o.	CD	0.38	0.28 - 0.5	**< 0.001**	0.21	0.14 - 0.28	**< 0.001**	0.29	0.13 - 0.54	**< 0.001**	0.54	0.41 - 0.66	**< 0.001**	0.50	0.23 - 0.77	**< 0.001**
IL-6 o.	UC	0.36	0.26 - 0.46	**< 0.001**	0.11	0.05 - 0.16	**< 0.001**	0.36	0.18 - 0.55	**< 0.001**	0.42	0.29 - 0.55	**< 0.001**	0.51	0.24 - 0.75	**< 0.001**
sIL-6R	CD	1.00	-3 - 7	0.37	5.00	2.5 - 9	**0.002**	3.00	0 - 5	**0.044**	0.00	-4 - 6	1	2.00	-3 - 6	0.36
sIL-6R	UC	4.00	1 - 10	**0.006**	4.00	1.5 - 9	**< 0.001**	1.00	-3 - 5	0.51	5.00	1 - 11	**0.004**	1.00	-3 - 5	0.47
sgp130	CD	15.50	1 - 33	**0.024**	10.50	-5.5 - 23	0.16	2.00	-11 - 19	0.77	15.50	-12 - 46	0.19	8.00	-14 - 40	0.57
sgp130	UC	32.50	-18 - 49	0.092	-20.00	-51 - 26	0.17	18.00	-26 - 36	0.23	23.50	-22 - 50	0.18	27.00	-34 - 41	0.13

All data except for small *p* values were rounded to the second decimal place.
Bold font, *p* value < 0.05.
95% CI, 95% confidence interval; CD, Crohn's disease; Dis., disease; Est., estimate; IL-6 o., ordinal data for interleukin-6; sgp130, soluble glycoprotein 130; sIL-6R, soluble IL-6 receptor; UC, ulcerative colitis.

Supplementary Table 7: Statistical data for Figure 3

		Active vs. controls			Inactive vs. controls			Inactive vs. active		
**Variable**	**Disease**	**Estimate**	**95% CI**	***p* value**	**Estimate**	**95% CI**	***p* value**	**Estimate**	**95% CI**	***p* value**
IL-6 ordinal	CD	0.58	0.46 - 0.67	**< 0.001**	0.13	0.07 - 0.19	**< 0.001**	0.42	0.26 - 0.59	**< 0.001**
IL-6 ordinal	UC	0.62	0.5 - 0.73	**< 0.001**	0.04	-0.01 - 0.09	0.072	0.62	0.4 - 0.8	**< 0.001**
sIL-6R	CD	3.00	0 - 8	**0.016**	3.00	0 - 8	**0.03**	0.00	-2 - 2	0.96
sIL-6R	UC	4.00	0 - 9.5	**0.024**	5.00	2 - 9	**< 0.001**	0.00	-2 - 3	0.86
sgp130	CD	6.50	-6 - 19	0.32	15.50	2 - 28	**0.022**	3.00	-11 - 21	0.68
sgp130	UC	11.50	-41 - 46	0.63	12.50	-10 - 45	0.19	-12.00	-29 - 5	0.44

All data except for small *p* values were rounded to the second decimal place.
Bold font, *p* value < 0.05.
95% CI, 95% confidence interval; CD, Crohn’s disease; IL-6, interleukin-6; sgp130, soluble glycoprotein 130; sIL-6R, soluble IL-6 receptor; UC, ulcerative colitis.

Supplementary Table 8: Statistical data for Figure 4

		**Active vs. controls**			**Inactive vs. controls**			**Inactive vs. active**		
**Variable**	**Disease**	**Estimate**	**95% CI**	***p* value**	**Estimate**	**95% CI**	***p* value**	**Estimate**	**95% CI**	***p* value**
IL-6 ordinal	CD	0.36	0.22 - 0.5	**< 0.001**	0.18	0.06 - 0.31	**< 0.001**	0.32	0.12 - 0.57	**< 0.001**
IL-6 ordinal	UC	0.42	0.22 - 0.61	**< 0.001**	0.15	0.02 - 0.29	**0.02**	0.42	0.16 - 0.73	**< 0.001**
sIL-6R	CD	3.00	-1.5 - 8	0.15	1.50	-3 - 7	0.51	2.00	-2 - 4	0.22
sIL-6R	UC	2.50	-2.5 - 11	0.41	5.00	1 - 10	**0.038**	1.00	-5 - 5	0.78
sgp130	CD	14.50	-20 - 26	0.33	5.50	-19 - 20	0.82	12.00	-30 - 50	0.56
sgp130	UC	-10.50	-68 - 100	0.85	-5.50	-99 - 20	0.61	n/a	n/a	n/a

All data except for small *p* values were rounded to the second decimal place.
Bold font, *p* value < 0.05.
95% CI, 95% confidence interval; CD, Crohn’s disease; IL-6, interleukin-6; n/a, not applicable; sgp130, soluble glycoprotein 130; sIL-6R, soluble IL-6 receptor; UC, ulcerative colitis.

Supplementary Table 9: Statistical data for Figure 5

		**Active vs. controls**			**Inactive vs. controls**			**Inactive vs. active**		
**Variable**	**Disease**	**Estimate**	**95% CI**	***p* value**	**Estimate**	**95% CI**	***p* value**	**Estimate**	**95% CI**	***p* value**
IL-6 ordinal	CD	0.64	0.51 - 0.77	**< 0.001**	0.22	0.1 - 0.34	**< 0.001**	0.44	0.24 - 0.66	**< 0.001**
IL-6 ordinal	UC	0.66	0.5 - 0.8	**< 0.001**	0.03	-0.04 - 0.12	0.43	0.63	0.36 - 0.85	**< 0.001**
sIL-6R	CD	5.00	1 - 8.5	**0.012**	5.00	1 - 12	**0.006**	-1.00	-5 - 2	0.33
sIL-6R	UC	1.00	-2 - 7	0.37	3.00	-1 - 9	0.13	-1.00	-4 - 2	0.48
sgp130	CD	14.50	-2 - 37	0.068	12.50	-2 - 34	0.074	2.50	-22 - 26	0.65
sgp130	UC	6.00	-47 - 66	0.86	11.50	-11 - 66	0.12	-5.50	-36 - 4	0.78

All data except for small *p* values were rounded to the second decimal place.
Bold font, *p* value < 0.05.
95% CI, 95% confidence interval; CD, Crohn’s disease; IL-6, interleukin-6; sgp130, soluble glycoprotein 130; sIL-6R, soluble IL-6 receptor; UC, ulcerative colitis.

Supplementary Table 10: Association of ordinal interleukin-6 levels with general, clinical and laboratory parameters (Figure 6)

Data group	Parameter	Crohn's disease (CD)			Ulcerative colitis (UC)		
		**OR**	**95% CI**	**P-value**	**OR**	**95% CI**	**P-value**
General data	Age at sampling [y]	0.51	0.20 - 1.31	0.15	1.23	0.50 - 3.03	0.66
	Allergy/asthma	1.72	0.45 - 6.49	0.43	0.22	0.04 - 1.35	0.079
	Appendectomy	0.28	0.06 - 1.41	0.11	0.55	0.06 - 5.46	0.61
	Arthritis as a comorbidity	1.02	0.48 - 2.17	0.96	0.58	0.23 - 1.47	0.23
	Body mass index (BMI) high (BMI ≥ 25) or normal (18.5 ≤ BMI < 25)	0.88	0.35 - 2.19	0.78	0.74	0.32 - 1.72	0.49
	Body mass index low (< 18.5) or normal (18.5 ≤ BMI < 25)	1.63	0.38 - 7.08	0.51	18.24	0.65 - 511.66	0.052
	Food allergy	0.88	0.25 - 3.11	0.84	0.48	0.03 - 6.54	0.57
	Gender	0.76	0.28 - 2.07	0.59	1.14	0.45 - 2.91	0.78
	Positive family history of inflammatory bowel diseases	0.41	0.13 - 1.30	0.13	0.87	0.27 - 2.87	0.82
	Smoker (current)	0.70	0.27 - 1.82	0.46	0.96	0.23 - 4.06	0.96
	Smoker (former or current)	1.43	0.58 - 3.53	0.44	1.40	0.53 - 3.66	0.49
	Surgery on gastrointestinal tract	0.56	0.23 - 1.39	0.2	1.66	0.53 - 5.18	0.38
	Time after diagnosis [y]	1.00	0.40 - 2.52	1	0.58	0.23 - 1.47	0.25
Activity (CD)	**Crohn's disease activity index (CDAI)**	**4.18**	**2.07 - 8.43**	**2.2e-05**	n/a	n/a	n/a
	**Harvey-Bradshaw Index (HBI)**	**4.28**	**2.13 - 8.59**	**1.2e-05**	n/a	n/a	n/a
Activity (UC)	**Colitis activity index (CAI)**	n/a	n/a	n/a	**2.98**	**1.53 - 5.80**	**0.0011**
	**Mayo Score**	n/a	n/a	n/a	**3.27**	**1.32 - 8.06**	**0.0099**
	**Partial Mayo Score (PMS)**	n/a	n/a	n/a	**8.61**	**3.92 - 18.95**	**1.5e-09**
Laboratory data	**Alanine transaminase (ALT) [U/L]**	**0.28**	**0.14 - 0.54**	**7.7e-05**	**0.44**	**0.22 - 0.89**	**0.019**
	Aspartate transaminase (AST) [U/L]	0.60	0.33 - 1.09	0.092	0.82	0.43 - 1.56	0.54
	**C-reactive protein (CRP) [mg/L]**	**22.53**	**10.39 - 48.85**	**7e-21**	**46.27**	**16.16 - 132.51**	**9e-24**
	Creatinine [mg/dL]	1.20	0.63 - 2.29	0.58	0.83	0.38 - 1.83	0.65
	**Haematocrit [vol%]**	**0.28**	**0.14 - 0.54**	**0.00014**	**0.30**	**0.15 - 0.59**	**0.0003**
	**Haemoglobin [g/dL]**	**0.27**	**0.14 - 0.53**	**0.00012**	**0.34**	**0.17 - 0.67**	**0.0015**
	**Leukocytes [/nL]**	**5.34**	**2.74 - 10.38**	**1.1e-07**	**4.12**	**2.06 - 8.24**	**2.1e-05**

	Soluble glycoprotein 130 [ng/mL]	1.46	0.57 - 3.73	0.43	0.82	0.19 - 3.51	0.79
	Soluble interleukin-6 receptor [ng/mL]	1.05	0.54 - 2.05	0.87	0.81	0.40 - 1.62	0.55
	**Thrombocytes [/nL]**	**4.72**	**2.43 - 9.15**	**3.1e-06**	**3.82**	**1.87 - 7.81**	**0.00021**
Medication	**Aminosalicylate therapy**	0.56	0.13 - 2.36	0.42	**0.12**	**0.03 - 0.56**	**0.0036**
	Immunosuppressive therapy	0.68	0.16 - 2.86	0.59	1.89	0.50 - 7.14	0.34
	Monoclonal antibody therapy, except for tumour necrosis factor-alpha (TNF-α) inhibitors	0.57	0.19 - 1.77	0.33	1.82	0.41 - 8.10	0.43
	Steroid therapy	1.31	0.46 - 3.75	0.62	1.12	0.31 - 4.09	0.86
	**TNF-α inhibitor therapy**	**0.21**	**0.21 - 0.21**	**0.014**	0.97	0.43 - 2.20	0.95

Bold font, significantly correlated ($p < 0.05$).
CI, confidence interval; n/a, not applicable; OR, odds ratio.

Supplementary Table 11: Association of soluble interleukin-6 receptor levels with general, clinical and laboratory parameters (Figure 6)

Data group	Parameter	Crohn's disease (CD)				Ulcerative colitis (UC)			
		**Beta**	**95% CI**	**R^2**	**P-value**	**Beta**	**95% CI**	**R^2**	**P-value**
General data	Age at sampling [y]	1.09	-1.38 - 3.56	0.0021	0.39	2.11	-0.93 - 5.14	0.0087	0.17
	**Allergy/asthma**	**-3.83**	**-6.53 - -1.14**	**0.0083**	**0.0054**	2.59	-1.38 - 6.56	0.0036	0.2
	Appendectomy	1.65	-1.83 - 5.13	0.0018	0.35	4.46	-3.16 - 12.08	0.0076	0.25
	Arthritis as a comorbidity	0.10	-1.30 - 1.50	1.7e-05	0.89	1.03	-1.29 - 3.36	0.0019	0.38
	**Body mass index (BMI) high (BMI ≥ 25) or normal (18.5 ≤ BMI < 25)**	**3.75**	**1.56 - 5.95**	**0.025**	**0.00084**	**3.79**	**1.37 - 6.21**	**0.029**	**0.0023**
	Body mass index low (< 18.5) or normal (18.5 ≤ BMI < 25)	1.90	-0.72 - 4.51	0.0051	0.15	0.12	-6.15 - 6.38	6.9e-06	0.97
	Food allergy	1.75	-0.71 - 4.21	0.0024	0.16	-1.68	-8.15 - 4.79	0.00047	0.61
	Gender	-0.92	-3.98 - 2.15	0.0014	0.56	0.95	-2.23 - 4.13	0.0018	0.56
	Positive family history of inflammatory bowel diseases	-0.34	-3.94 - 3.26	0.00017	0.85	-1.16	-5.27 - 2.96	0.0014	0.58
	Smoker (current)	-0.55	-3.22 - 2.12	0.00049	0.69	-3.96	-8.77 - 0.85	0.0092	0.11
	Smoker (former or current)	0.58	-1.77 - 2.94	0.00061	0.63	0.59	-2.66 - 3.84	0.00064	0.72
	**Surgery on gastrointestinal tract**	**2.98**	**0.77 - 5.20**	**0.015**	**0.0084**	0.64	-2.86 - 4.14	0.00031	0.72
	Time after diagnosis [y]	1.33	-1.08 - 3.74	0.0031	0.28	0.48	-2.49 - 3.45	0.00041	0.75
Activity (CD)	Crohn's disease activity index (CDAI)	0.93	-0.27 - 2.14	0.0015	0.13	n/a	n/a	n/a	n/a
	Harvey-Bradshaw Index (HBI)	0.43	-0.74 - 1.60	0.00033	0.47	n/a	n/a	n/a	n/a
Activity (UC)	Colitis activity index (CAI)	n/a	n/a	n/a	n/a	0.30	-1.41 - 2.00	0.00018	0.73
	Mayo Score	n/a	n/a	n/a	n/a	0.75	-1.61 - 3.10	0.0011	0.53
	Partial Mayo Score (PMS)	n/a	n/a	n/a	n/a	0.05	-1.68 - 1.78	5.1e-06	0.95
Laboratory data	**Alanine transaminase (ALT) [U/L]**	**2.55**	**1.43 - 3.67**	**0.012**	**9.2e-06**	1.27	-0.44 - 2.98	0.0032	0.14
	**Aspartate transaminase (AST) [U/L]**	**1.23**	**0.18 - 2.29**	**0.0027**	**0.022**	0.64	-0.99 - 2.26	0.00081	0.44
	**C-reactive protein (CRP) [mg/L]**	**1.66**	**0.45 - 2.87**	**0.005**	**0.0072**	0.03	-1.62 - 1.67	1.2e-06	0.98
	Creatinine [mg/dL]	0.76	-0.46 - 1.98	0.001	0.22	-1.38	-3.50 - 0.74	0.0038	0.2

	Haematocrit [vol%]	-0.53	-1.87 - 0.81	0.00051	0.43	0.08	-1.64 - 1.80	1.3e-05	0.93
	Haemoglobin [g/dL]	0.27	-1.06 - 1.61	0.00013	0.69	0.49	-1.34 - 2.32	0.00047	0.6
	Interleukin-6 [pg/mL]	0.36	-0.13 - 0.85	0.0011	0.15	-0.31	-1.04 - 0.42	0.0009	0.41
	**Leukocytes [/nL]**	**1.20**	**0.11 - 2.29**	**0.0026**	**0.031**	1.35	-0.21 - 2.91	0.0036	0.089
	**Soluble glycoprotein 130 [ng/mL]**	**3.95**	**2.57 - 5.32**	**0.032**	**3.6e-08**	**6.31**	**1.90 - 10.73**	**0.062**	**0.0057**
	Thrombocytes [/nL]	0.89	-0.40 - 2.17	0.0014	0.18	0.56	-1.44 - 2.57	0.00063	0.58
Medication	Aminosalicylate therapy	0.99	-3.04 - 5.03	0.0011	0.63	2.97	-1.59 - 7.52	0.0059	0.2
	Immunosuppressive therapy	-2.94	-6.82 - 0.93	0.0072	0.14	-2.19	-6.19 - 1.81	0.0057	0.28
	**Monoclonal antibody therapy, except for tumour necrosis factor-alpha (TNF-α) inhibitors**	-0.68	-3.15 - 1.80	0.00043	0.59	**-4.08**	**-7.83 - -0.32**	**0.0042**	**0.034**
	Steroid therapy	0.93	-1.64 - 3.50	0.0017	0.47	2.20	-1.32 - 5.72	0.0078	0.22
	TNF-α inhibitor therapy	1.11	-1.24 - 3.46	0.0011	0.35	-1.45	-3.60 - 0.71	0.0043	0.19

Bold font, significantly correlated ($p < 0.05$).
CI, confidence interval; n/a, not applicable.

Supplementary Table 12: Association of soluble glycoprotein 130 levels with general, clinical and laboratory parameters (Figure 6)

**Data group**	**Parameter**	**Crohn's disease (CD)**				**Ulcerative colitis (UC)**			
		**Beta**	**95% CI**	**R^2**	**P-value**	**Beta**	**95% CI**	**R^2**	**P-value**
General data	**Age at sampling [y]**	**17.61**	**2.89 - 32.33**	**0.038**	**0.019**	21.99	-20.94 - 64.93	0.033	0.3
	Allergy/asthma	-10.95	-33.63 - 11.73	0.0056	0.34	79.53	-36.92 - 195.97	0.022	0.17
	Appendectomy	22.07	-4.99 - 49.13	0.021	0.11	1.24	-71.44 - 73.92	5.1e-05	0.97
	Arthritis as a comorbidity	-3.73	-13.36 - 5.90	0.0017	0.45	24.18	-11.14 - 59.50	0.031	0.18
	Body mass index (BMI) high (BMI ≥ 25) or normal (18.5 ≤ BMI < 25)	-4.93	-19.75 - 9.88	0.0031	0.51	19.05	-12.50 - 50.59	0.032	0.23
	Body mass index low (< 18.5) or normal (18.5 ≤ BMI < 25)	1.84	-17.21 - 20.89	0.00022	0.85	-31.36	-88.84 - 26.12	0.017	0.27
	Food allergy	11.98	-5.23 - 29.19	0.0082	0.17	Only one characteristic value			
	Gender	-4.47	-22.24 - 13.30	0.0023	0.62	-33.86	-73.53 - 5.80	0.092	0.09
	Positive family history of inflammatory bowel diseases	2.92	-17.61 - 23.45	0.00084	0.78	Only one characteristic value			
	Smoker (current)	-7.41	-23.80 - 8.97	0.0061	0.37	Only one characteristic value			
	**Smoker (former or current)**	12.41	-3.88 - 28.69	0.019	0.13	**55.43**	**20.76 - 90.10**	**0.24**	**0.0033**
	**Surgery on gastrointestinal tract**	**24.75**	**10.50 - 39.00**	**0.071**	**0.00071**	41.51	-9.45 - 92.48	0.09	0.1
	Time after diagnosis [y]	-8.62	-24.85 - 7.62	0.0092	0.3	10.81	-32.81 - 54.43	0.0083	0.61
Activity (CD)	**Crohn's disease activity index (CDAI)**	**9.96**	**1.28 - 18.64**	**0.013**	**0.025**	n/a	n/a	n/a	n/a
	**Harvey-Bradshaw Index (HBI)**	**10.69**	**2.13 - 19.25**	**0.015**	**0.015**	n/a	n/a	n/a	n/a
Activity (UC)	Colitis activity index (CAI)	n/a	n/a	n/a	n/a	3.96	-17.35 - 25.28	0.0012	0.71
	Mayo Score	n/a	n/a	n/a	n/a	-15.61	-54.09 - 22.87	0.018	0.41
	Partial Mayo Score (PMS)	n/a	n/a	n/a	n/a	-2.15	-25.60 - 21.30	0.0004	0.85
Laboratory data	**Alanine transaminase (ALT) [U/L]**	**11.71**	**3.85 - 19.58**	**0.017**	**0.0036**	**31.74**	**10.94 - 52.53**	**0.075**	**0.0033**
	**Aspartate transaminase (AST) [U/L]**	**9.07**	**1.82 - 16.32**	**0.011**	**0.014**	**21.69**	**3.80 - 39.59**	**0.041**	**0.018**
	C-reactive protein (CRP) [mg/L]	2.28	-5.88 - 10.45	0.00065	0.58	-7.21	-29.00 - 14.57	0.0042	0.51
	Creatinine [mg/dL]	3.96	-4.17 - 12.10	0.002	0.34	-2.09	-31.66 - 27.48	0.00025	0.89

	**Haematocrit [vol%]**	**-11.70**	**-20.60 - -2.80**	**0.017**	**0.01**	2.07	-18.75 - 22.89	0.00035	0.84
	Haemoglobin [g/dL]	-4.82	-14.16 - 4.53	0.0029	0.31	4.26	-18.21 - 26.74	0.0015	0.71
	**Interleukin-6 [pg/mL]**	**4.08**	**0.74 - 7.42**	**0.011**	**0.017**	-1.89	-10.08 - 6.30	0.0015	0.65
	Leukocytes [/nL]	1.95	-5.56 - 9.47	0.00048	0.61	0.97	-17.93 - 19.88	7.1e-05	0.92
	**Soluble interleukin-6 receptor [ng/mL]**	**27.20**	**18.92 - 35.47**	**0.085**	**4e-10**	**26.06**	**5.16 - 46.97**	**0.057**	**0.015**
	Thrombocytes [/nL]	-2.82	-11.65 - 6.01	0.001	0.53	13.58	-10.38 - 37.54	0.015	0.26
Medication	Aminosalicylate therapy	-0.49	-22.23 - 21.25	2.4e-05	0.96	3.70	-36.08 - 43.48	0.0008	0.85
	Immunosuppressive therapy	8.39	-17.28 - 34.07	0.005	0.52	7.36	-46.82 - 61.53	0.0034	0.78
	Monoclonal antibody therapy, except for tumour necrosis factor-alpha (TNF-α) inhibitors	-5.25	-28.16 - 17.65	0.0018	0.65	Only one characteristic value			
	Steroid therapy	-1.69	-16.90 - 13.51	0.00043	0.83	-12.28	-50.89 - 26.32	0.011	0.52
	TNF-α inhibitor therapy	0.33	-15.83 - 16.48	7.6e-06	0.97	-9.74	-38.69 - 19.21	0.0069	0.5

Bold font, significantly correlated ($p < 0.05$).
CI, confidence interval; n/a, not applicable.

Supplementary Table 13: Associations of interleukin-6, soluble interleukin-6 receptor and soluble glycoprotein 130 levels with disease location

		**IL-6 (ordinal data)**		**sIL-6R**		**sgp130**	
**Disease**	**Location**	**OR**	***p* value**	**Effect est.**	***p* value**	**Effect est.**	***p* value**
CD	Stomach to proximal ileum affected	1.65	0.36	1.11	**0.002**	0.18	**< 0.001**
CD	Terminal ileum affected	3.45	**0.016**	-1.34	**0.003**	-2.67	**< 0.001**
CD	Colon affected	1.86	0.13	-1.32	**0.002**	-16.63	**0.019**
UC	Only rectum and sigma affected	1.30	0.82	1.90	0.43	-66.97	0.32
UC	Left-sided colitis (up to splenic flexure)	6.27	0.09	1.56	0.51	-29.04	0.57
UC	Subtotal colitis (up to hepatic flexure)	7.33	0.09	2.72	0.33	-47.67	0.48
UC	Pancolitis	5.54	0.10	0.74	0.74	-39.69	0.41

All data except for small *p* values were rounded to the second decimal place.
Bold font, *p* value < 0.05.
CD, Crohn’s disease; Effect est., estimated effect size; IL-6, interleukin-6; OR, odds ratio; sgp130, soluble glycoprotein 130; sIL-6R, soluble IL-6 receptor; UC, ulcerative colitis.
